# Resourcefulness, Desperation, Shame, Gratitude and Powerlessness: Common Themes Emerging from A Study of Food Bank Use in Northeast Scotland

**DOI:** 10.3934/publichealth.2015.3.297

**Published:** 2015-07-23

**Authors:** Flora Douglas, Jennifer Sapko, Kirsty Kiezebrink, Janet Kyle

**Affiliations:** 1 Rowett Institute of Nutrition and Health, University of Aberdeen, AB25 2ZD, Scotland; 2 Institute of Applied Health Sciences, University of Aberdeen, Scotland

**Keywords:** food poverty, food banks, deprivation, Scotland, nutrition, mental well-being, policymaking, qualitative research, mixed methods

## Abstract

There is growing policy maker and public concern about current trends in food bank use in Scotland. Yet little is known about the experiences of those seeking help from food banks in this country. This research aimed to address this issue by studying the use and operation of a food bank situated in a rich northeast city during January and June 2014. The study aimed to establish who was seeking help from the food bank, their reasons for doing so, and what those who did thought of, and dealt with the food they received from it. Consequently, an audit of the food bank's client database, four months of participant observation based in the food bank, and seven face-to-face interviews with current and former food bank clients were conducted. The audit revealed that clients came from a range of socio-economic backgrounds, with men more likely to access it compared to women. Debt and social security benefit delays were cited as the main reasons for doing so. Qualitative data confirmed that sudden and unanticipated loss of income was a key driver of use. Resourcefulness in making donated food last as long as possible, keeping fuel costs low, and concern to minimise food waste were commonly described by participants. Desperation, gratitude, shame and powerlessness were also prevalent themes. Furthermore, clients were reluctant to ask for food they normally ate, as they were acutely aware that the food bank had little control over what it was able offer. Insights from this study suggest that recent UK policy proposals to address food poverty may have limited impact, without concomitant effort to address material disadvantage. Research is urgently required to determine the precise nature and extent of household level food insecurity in Scotland, and to consider monitoring for adverse physical and mental health outcomes for those affected by it.

## 1. Introduction

Food poverty/insecurity has attracted considerable media, political and public health interest in recent years, and has become the subject of policy, civic and political concern in Scotland and the UK [1]–[6]. The appearance of so-called “food banks”––charitable emergency feeding centers––has been the subject of much of this attention [4]. The UK has a long tradition of charitable giving and supporting people experiencing crisis or destitution. However, it is the unprecedented rise in the number of organizations setting up to provide help to people to feed themselves and their families, and, in the number of people seeking help from them, that has attracted recent attention to the issue [7]. Food banks are described as *‘voluntary community organizations that solicit food and financial donations from the public and corporate sectors and distribute food assistance locally according to whatever principles they have established’* according to Canadian food security scholar Valerie Tarasuk [8]. In the UK food banks are provisioned from public and cooperate food donations, but are also sometimes able to access food donations through a national network called Fareshare; a charity that sources and distributes waste or surplus food from the food and drink industry to organizations concerned with addressing need [7]. While it has not been possible to establish the precise number and range of food banks that exist in the UK (despite efforts to do so), the Trussell Trust has become the best-known food bank operator in this country. However, a recent report published in response to the UK All Party Parliamentary inquiry into hunger and food poverty acknowledged that while the Trussell Trust is the most commonly known and visible food bank network in the UK, it is probably only representative of about half the food banks currently operating here [9]. At the time of writing, 420 Trussell Trust food banks were said to be operating in the UK (ibid). Those other food bank providers have emerged either from organizations that have established histories of community-based food work, or, are completely new initiatives that have organically emerged from local communities themselves in response to the current context [7].

Recent research has indicated that food banks operate on the basis of different philosophies and practices; some better described than others. For example, the Trussell Trust is a faith-based (but non-proselytising) enterprise that offers three days' worth of food and receipt of a voucher (issued to a person or household by a health and social care-referring agency), to a maximum of three a year [10], while others employ different eligibility criteria and provision practices [7]. What is clear is that the numbers of people here seeking help from charitable organisations distributing donated food to people for free has markedly increased over the last few years [10]. During 2013/14, it was estimated that over 20 million meals were distributed to people living in the UK by charitable organisations [1]. There was a recorded two-fold increase in numbers of Trussell Trust food banks operating in the UK in the 12 months from October 2012, and a three-fold increase in the numbers of people seeking emergency food aid from them at that time.

There is growing concern that such stark increases in food bank use is evidence of an emerging public health crisis and of acute need within the UK context, and requires a different policy approach to address it [3],[11]–[14]. However, again, the picture is not clear, as household level food insecurity data is not collected in this country [15]. This phenomenon is considered to be due in part to the fact that the traditional UK policy view of food security (or insecurity) has focused on efficiency in the retail and employment markets, and has regarded individual and household food consumption to be a private matter [8],[16]. In addition, food bank data systems (such as they exist) do not monitor household conditions or practices. There is also a dearth of contemporary qualitative data derived from the direct experiences of those who are seeking emergency food aid or who are food insecure in the UK and Scotland that could be used to inform policy in the Scottish jurisdiction.

This paper addresses that knowledge gap by providing some insight into the experience of food bank use by presenting the results of a mixed methods study conducted with volunteers and individuals using a non-russell Trust community food bank in the north east of Scotland. In particular it provides some present day insights into the lived experience of people's use of a food bank in one of the most affluent cities in the UK. Due to pre-existing links between an independent local community food programmed and the University, the study aims and design were collaboratively developed and agreed between the authors and the local community food programme manager.

## 2. Materials and methods

The study aimed to establish what factors had caused individuals to seek food aid from the food bank; the strategies food bank clients were using to try to reduce or mitigate household food insecurity in the current economic context; what they thought of the food they received from the food bank; and how they were incorporating it in their diets. Additionally the study describes a client database audit to establish the demographic profile of people who had accessed the food bank during January and April 2014 using data routinely collected by the food bank.

### 2.1. Client database audit

The food bank maintains a client database to record use and track trends in use over time. This database is populated with data derived from a standardized questionnaire that is issued to and completed by each client using the food bank at the point of access. The questionnaire included basic demographic details (gender, date of birth, marital status, number of dependents). Additional data was recorded about reasons for accessing service, route of referral, additional service requirements (e.g., debt management, health concerns). All records for January – April 2014 were checked by the food bank for duplications and anonymised before being made available to the research team. Using the Scottish Index for Multiple Deprivation (SIMD) [17] participant was assigned a score (1 to 10, 1 being most deprived 10 least) based on their postcode area of residence. Where clients had changed address within the time period of the study, the average SIMD score was calculated based on all locations that they had registered. For those cases where they had no permanent accommodation (such as staying temporarily with friends or were homeless), a SIMD score of one was assigned irrespective of the SIMD for the postcode given.

### 2.2. Participant observation and semi-structured interviews

The qualitative element of the study was conducted using principles and techniques found in Grounded Theory and ethnographic approaches [18],[19]. As researchers, we were very aware of the difficult nature of the research we were undertaking in trying to gain a picture of the lived experiences of food bank clients. We imagined that talking about the difficulties faced by those who are struggling to feed themselves or their family would be hard to do with a complete stranger. We imagined that this would be a hard-to-reach group from an academic's position, or an outsider's perspective [20],[21]. We were also concerned to ensure that potential participants were approached with respect and sensitivity, mindful that the encounter could be potentially emotionally damaging for people who may already be feeling stigmatized by their having to use a food bank. Consequently, the second author (JS) spent four months in the food bank as a participant observer researcher in order to develop an in-depth understanding of the day-to-day dynamics and processes of the food bank, and develop the rapport and trust required to engage with volunteers and people using the food bank.

Ethics permission was sought and granted by the college ethics review board, application no CERB/2014/4/1047.

In-depth interviews were conducted with current and former food bank users towards the end of the four-month observational study. A combination of purposive and convenience sampling was used to identify potential participants who were invited to take part in a formal in-depth interview. This proceeded through a staged process of recruitment. Once the client database audit was completed and the demographic profile of food bank users was established, the research field worker identified those individuals who broadly reflected that profile, and that she had come to know through the observation period. She then approached and asked potentially eligible participants to take part in a formal interview. Those who indicated they were interested in taking part were then provided with written or audio information about the study and written consent was secured before a formal interview took place, with assurances that their anonymity would be protected.

A topic guide was used to guide the discussions and enabled the researchers to combine inductive and deductive reasoning congruent with Grounded Theory approaches to generate and analyse the data [18]. Topics explored during the interviews included: a. an exploration of factors that led participants to approach the food bank for help; b. participants' normative food preferences; c. their perspectives about current food choices and meal practices; and d. their views about food they had received from the food bank, and about food banks in general. Interviews were conducted on the food bank premises and lasted between 15–20 minutes. At the conclusion of the interview, participants received a £5 voucher in recognition of the time they given to the study. They were unaware that they would receive this recompense until that point. All interviews were audio-recorded and transcribed verbatim.

The data were manually analyzed thematically [22]. Initially a sample of interview transcripts were read and re-read independently by two researchers (FD & JS) to identify the key concepts and themes and a draft coding index was drawn up. The researchers met to discuss their initial analysis: areas of difference were identified and areas of disagreement were resolved. Constant comparison method was used throughout to ensure consistency of coding and assigning of coded data to the emergent themes and categories, and, to ensure that possible new themes were not being overlooked. Disconfirming data was also sought within the data set. Anonymised, illustrative quotes are used in the results section to illustrate the key themes.

## 3. Results

### 3.1. Study context

The food bank that was the focus of this study had been a recent addition to the operation of a long-standing, community-based social enterprise, which is situated in the centre of the largest city in the northeast of the country. This secular programme was first established in 1997, with the initial aim of improving the health and well-being of disadvantaged communities living in the north east of Scotland. During its previous 15 years existence, the programme had provided a range of food-based services; including a low-cost retailing service for low-income communities, and food-related work intended to enable long-term unemployed individuals with a background of mental health problems or offending behavior, gain work experience. The food bank operation opened in 2012 and was situated in the central business district of the city, close to popular retail and restaurant areas, which was also close to the city's industrial harbor area.

The food bank was open to clients five days a week from 10 am till 4 or 5 pm, depending on food and staff availability. The food bank was managed by a paid staff member, but was reliant on volunteer labor for the bulk of its day-to-day operations. Some, but not all of the volunteers were also former food bank clients

The food bank was provisioned in a number of ways, including public and corporate food donations, bulk buying of food from periodic, successful grant funding, and through participation in the afore mentioned Fare share scheme. Over the period of the fieldwork, it became obvious that the food bank had little control over what food items it received, how much food it received or when it would arrive. It was noticeable that the food items donated to it varied from week to week, in terms of both nutritional quality and quantity. Goods were also invariably almost out of date when they arrived at the food bank. This meant that if clients did not happen to come in on the days fresh foods were available, their choices were often limited to items that had a longer shelf life.

Individuals could be referred to the food bank by local health or social care providers, or, could refer themselves to it directly. Clients were generally allowed to use the food bank once a week, but some were able to access it more often than this for reasons that were not always clear to the researcher. People referred by social or health care workers were sometimes in possession of a pre-paid card that allowed them to purchase very low price food items that were also available for sale from the food bank. People without those cards were given a food parcel. In general, the parcel (bag) given to each client variously contained some type of carbohydrate (pasta, rice or cereal), a boxed drink, packaged or tinned produce (i.e., baked beans, soups, sauces), tea, sugar, any additional items that the food bank had available to them at any given time. It was also often supplemented with additional short shelf life produce of fresh fruit, vegetables, milk and bread as described above. These food items were usually made available to clients on a first come, first served basis.

### 3.2. Audit findings

It is important to note that the database contained a lot of missing data particularly so when considering marital status and reported number of household dependants. The majority of recorded users claimed single status. Based on the Scottish Index of Multiple Deprivation as a marker for socio-economic status, it was interesting to note that food bank clients were drawn from across the deprivation spectrum, albeit unevenly so. Men were also more likely to use the food bank compared to women. The majority of users had self-referred, and debt and benefit delays cited as the main reasons for approaching the food bank.

**Table 1.** Client Descriptives January 2014–April 2014.

	Number of clients (%)*n* = 423 (men 255, women 90, missing data 78)												
**Marital Status**	**Single**			**Married**			**Living with partner**			**Other**			**Missing Data**
	81(19.1)			3(0.7)			7(1.7)			4(0.9)			328(77.5)
**Age range**	**17–25**		**26–35**			**36–45**		**46–55**			**> 56**		
**(years**)	25(5.9)		71(16.8)			72(17.0)		41(9.7)			19(4.5)		195(46.1)
**Referral Agency**	**Council**		**Self**			**Social work**		**Welfare**			**Other**		
	36(8.5)		189(44.7)			16(3.8)		8(1.9)			28(6.6)		146(34.5)
**Referral reason**	**Debt**		**Delay in benefits**			**No money**		**Sanctioned DWP^a^**			**Other**		
	107(25.5)		118(27.9)			1(0.2)		33(7.8)			23(5.4)		141(33.3)
**SIMD^b^**	**1**	**2**	**3**		**4**	**5**	**6**	**7**	**8**		**9**	**10**	
	50(11.8)	87(20.6)	77(18.2)		22 (5.2)	21(5.0)	20(4.7)	18(4.3)	12(2.8)		8(1.9)	4(0.9)	104(24.6)

^a^ DWP – Department of works and pensions.

^b^ SIMD – Deprivation defined using Scottish index of multiple deprivation index, where 1= most deprived and 10 least deprived http://www.scotland.gov.uk/Topics/Statistics/SIMD.

### 3.3. Qualitative results

From the audit and fieldwork, twenty two people were identified as potential interviewees, and approached to take part in an interview. In the end, seven individuals participated in the interview study, five men and two women ranging from 25–50 years of age. One woman had two children under the age of 10 living with her. All were white and indigenous to the local area.

### 3.4. Factors leading to emergency food aid seeking

#### 3.4.1 Sudden financial shocks and changes to personal circumstances

Most commonly participants talked about a sudden change in their personal circumstances, such as losing a job, becoming homeless or becoming ill as the main factor that had driven them to seek help from the food bank. Reports of sudden and severe reductions in normal levels of income were also commonplace in participants' narratives and cited as the main reason for approaching the food bank. For example, one male participant (who was in his 40s) had worked offshore in the oil industry for years, had received a good income while working, and had never experienced financial problems before. He had recently experienced some health problems and needed to be hospitalised for those. Once out of the hospital, he had been made redundant from work due to ongoing uncertainty about his health. Having no savingswhen he became unemployed, his money had quickly dwindled and he became homeless. At the time of the study he was living in and out of hostels, and the local authority had directed him to the food bank for help. He talked about using it as little as possible and the shame he felt at not being self-sufficient. He had been finding it hard to find employment due to his continuing health problems, which had been making it difficult for him to work continuously. Another participant had also lost his job and had rapidly run out of money. He had applied for unemployment benefit but had not managed to secure any income through this means due to administrative problems. Before approaching the food bank he had sought help for the Salvation Army where he had been offered help with budgeting, which he described thus:

*Help me budget, but I says to him, you can't help me budget cause I don't have a budget* P7

For participants normally in receipt of some form of state benefit, being subject to withdrawal or some form of benefit sanction commonly featured in their stories of seeking help. In one case, a £20 a week reduction in one participant's benefits had had a catastrophic effect on his household budget, meaning he had no money to buy food.

#### 3.4.2 Existing life circumstances

Recurring indebtedness also featured in the interviews. One male participant (in his 30s) had just got out of prison. He talked about having to borrow money while in prison, which he had to repay on release. This had caused him to use money intended for food and shelter (out of prison), to pay back that debt, leaving him destitute. He had subsequently found that having a prison record made it difficult to find work, and he had become homeless.

Challenging life circumstance issues commonly featured in participants' stories, including chronic ill health, having pervious or current drugs and alcohol dependency issues, being ex-service personnel, or ex-offenders. Another male participant deemed medically unfit to work explained he had been actively trying but failing to find work. His social security entitlements had been reduced in recent months (due to Government public spending cutbacks) and he was finding it hard to manage on this lower monthly income, and had no savings to fall back on.

### 3.5 Normal food preferences

Participants were asked what they normally like to eat when money was not a problem for them. Meals containing meat, which were described as meals containing beef and fish were most commonly mentioned. Food that was tasty was important, as was eating “proper meals” and food that “fills you up”. Home cooked food was also frequently referred to as the type of food participants preferred to eat when not in food crisis, illustrated by this quote:

*Oh I would want to eat, um, like proper meals, like you're getting the mince, my pork chops, know what I mean, tatties [potatoes] and veg. But also I like cakes and puddings, stuff like that as well* (P4)

Getting value for money and shopping around was also important to them when not in food crisis. Takeaway food featured as something enjoyed as a treat when money was not an issue. One participant also talked about realizing that the food and meals he normally ate were determined more by habit than he realized since he had had to think more about what and how to eat due being in food crisis.

Some participants described aspects of an underlying health condition when talking about their normal eating habits and food preferences. Those individuals those who disclosed they were suffering from a chronic health condition did talk about nutritional considerations when describing their food consumption patterns, as it related to their condition.

### 3.6 Current food choices and meal practices

#### 3.6.1 Compromised food choices

When participants were asked about their current food choices since seeking help from the food bank, we found that no participant was eating meals they normally ate or necessarily liked. The lack of meat in their diets was frequently mentioned as they thing that they were missing most. Tinned food, pasta, rice and vegetables were the food items they most commonly talked about were receiving and incorporating into their current daily food intake. Noticeably, all participants seemed accepting of this, rather than complaining about it.

#### 3.6.2 Transportation challenges

Some participants reported that their food parcel's weight presented them with challenges in transporting it back to their home or hostel. None of those interviewed owned or had access to a car, so everything had to be transported in carrier bags from the food bank. One male participant talked about this not being a particular problem for him (as a single man he observed) but something that he was concerned about for other people, especially women with young families.

#### 3.6.3 Cooking and food storing

It was also clear that some experienced further significant challenges in making the contents of the food parcel into palatable meals due to the food preparation and cooking resources that were available to them in the place where they were living. Those living in hostel accommodation or bed and breakfast accommodation found preparing and storing food was not straightforward and indeed for some, somewhat precarious. In the following passage, one participant provides some insight into that uncertainty when talking to the researcher. In this excerpt he explains when talking about his attempts to eat a balanced diet, that although he may well have received healthy food items from the food bank; as a homeless person reliant on shared hostel cooking facilities, this did not necessarily mean he is easily able to prepare meals with those items. He pointed out that he could not predict who was going to be in the hostel kitchen when it came time for him to cook a meal, or, that they would necessarily be happy to share those facilities with him. He indicated that he would sometimes modify his plans according to the dynamics that are taking place within the kitchen at the time:

P1 *I try to eat a balanced diet*.

Int Yea? How easy do you think that is for you to do?

P1 *Well they give ya a lot of rice and they give ya a lot of vegetables... but if you're homeless like me it is not necessarily easy to cook those types of food*.

Int Right. Do you not have the appliances to cook it?

P1 *Well, I do. I'm sharing the appliances in the hostel*.

Int OK

P1 *But um [quiet tone and pauses for a few seconds], it's not one hundred percent reliant ya know*.

Int Right, depends on?

P1 Well whoever is sharing the hostel you know. Sometimes you get good people and sometimes you get bad people. So you just go with the flow

Int Whoever gets it first kind of thing?

P1 Yea

Another participant talked about thinking about fuel costs when deciding what he did with the contents of his food bag. Here he explains his reticence to use the oven of his cooker thus:

What part of the cooker I'm using because the oven uses up a hellava of a lot of electricity. When you're on a budget and that you have to watch the electricity. So it's normally just boiled things or stewed things. P6

It was common to hear accounts or suggestions from participants that illustrated the thought given to preparing and cooking the food they had received. A few talked about ‘stretching out’ the food received so that it could be eaten the following day or days. For example, this participant describes his practice of preserving meat in a bottled curry sauce that he routinely received from the food bank:

What you can do is, and a lot of people don't realise it. See for your meat, even if you just got a fridge or something if you put salt and pepper on meat and then you put your tikka masala on top of it you can marinate it for 24 hours and then it will last for about 3 or 4 days. P1

#### 3.6.4 Dealing with unwanted food items

Another common theme running throughout all participants narratives was their concern to waste as little food as possible. A range strategies were being used to avoid wasting unwanted food, including making perishable food items, or those with a limited shelf life last as long as possible as described above. All participants talked about receiving food they did not like or found difficult to eat. However, rather than rejecting this unwanted food, it seems to have been taken by each participant and redistributed to others. Much of it had been given away to friends or acquaintances who they thought would be able to use it, illustrated here:

Cause there is a girl on the ground floor of any building with 3 kids and a dog. I get a lot of stuff I ask her she says you like rice, you like pasta, stuff like that. If I don't need it I give it to her. I won't throw it in the bin. P6

One participant talked about being able to use that surplus or unwanted food in exchange for being able to sleep on a friend's sofa at different times during the week. He talked about this in terms of informal rules that he, and others in a similar position to himself, adhered to avoid sleeping rough. He regularly sought a place to sleep at a different friend's house each week.

#### 3.6.5 Returning for more help only when necessary

Despite the fact that this food bank allowed clients to return once a week for as long as they needed help, returning to the food bank seems to be something that participants only did when home food supplies had been exhausted, and when they had no money to buy food. One man described only going back to the food bank once his benefit money had run out. Another talked about having to exist on £2.00 each week after all his bills and debts had been paid from his benefits.

#### 3.6.6 Nutritional considerations

We also asked participants about the extent to which nutritional concerns figured in their current diets. It was clear that securing and consuming some form of sustenance was the key consideration for most. However, one male participant, who did not refer to any ongoing health issue, also indicated that nutrition a key concern for him. Making his very low budget and the food he was able to buy or get access to through via the food bank last as long as possible, was his primary concern. He also described the process he engaged in day-to-day to try and achieve some sort of nutritional balance over the course of a few days, which he said he could not manage to achieve on a daily basis. He did this by deliberately inserting nutritionally different food items over the course of a few days to achieve this balance, highlighted here:

*Just basically that I know my body needs so much fat, it needs so much carbs, it needs so much, you know what I mean?..I know if I haven't got it, you know what I mean, to try to make up for it when I have got it, you know what I mean….So I do know the awareness of, you know what I mean, not to eat two boxes of cheese at once….I don't get my benefits till next week so I got to get a food parcel. Normally make that last… I normally do, eh, pasta and masala…And you know that veg that is there as well. I add that through it. So that's a couple of meals.* P4

Bearing this example in mind, it was not clear from the interviews that took place with those who disclosed an underlying health condition, if they felt they were having their nutritional needs met or not. In addition, no participants raised any issue of dietary intolerances such as gluten or lactose intolerances.

### 3.7 Seeking and using emergency food aid

While discussing their individual accounts of factors and circumstances that had led to our participants to approach the food bank, and in describing their normal (pre-crisis) food preferences and practices compared to their current food and meal choices and practices, a range of additional themes emerged.

#### 3.7.1 Desperation and shame

In recounting stories about their various journeys to the food bank, feelings of shame and desperation were evident:

*It's a very humbling experience, very embarrassing and you feel ashamed, but you're desperate.* P1

Desperation was also expressed it terms of descriptions of the food bank as a “lifeline”, and as something that prevented some individuals from shoplifting to feed themselves, or risking further terms in prison. There was a sense from the interviews, and from other people that the researcher had talked to during the fieldwork, that people had had nowhere else to turn. For example, one participant said he only approached the food bank after he had eaten nothing for 6 days.

One female participant regarded the food bank as a lifeline to a better way of life. From this person's perspective, it had represented a way back into work, as well as something that she needed to alleviate an immediate food crisis situation. She said she had deliberately approached the food bank because she thought it would provide her with a route into formal employment. She talked about knowing that she would be able to get voluntary work experience and had viewed it as opportunity to leave both her current economic and social situation. She was long term unemployed and been finding it impossible to get paid work. She had been alcohol dependent in the past, and had two young children who did not live with her. She described her work at the food bank keeping her away from friends and from the ‘bad situation’ she had been in before. At the same time she talked about the people she saw using the food bank being in a worse position than herself, and the food bank being a lifeline to them: i.e. a *lot of people would starve. A lot of people would go hungry. Cause there are a lot of people out there that need places like this*. She asserted that she would not have volunteered at the food bank, if she had not been able to do so, and would have ‘gone without’ instead. Yet we know from the field work that she was in receipt of food from the food bank. Therefore, these findings indicate that the food bank seems to have represented a ‘life line’ for desperate or distressed people in two distinct but overlapping ways: i.e. as a life line to assuage hunger and food crisis, and, as a means to a better way of life that was not readily accessible through formal paid employment.

#### 3.7.2 Gratitude and powerlessness

These expressions of shame and desperation co-existed with equally apparent themes of gratitude and powerlessness. Gratitude was expressed not only about the food received from the food bank, but also in relation to the support received from staff they encountered there. Effusive statements of gratitude were commonplace, highlighted here:

(Name of food bank manager) and *her workers are working fantastically, you know what I mean. They give ya what you need, you know what I mean. It's not just me, it's every customer I've come in and seen they've helped out.* P4

Linked to this gratitude was a strong sense of powerlessness. For example, almost all participants indicated that they did not like all the food items they received, but did not feel able to ask for what they normally ate or liked. Almost all talked about the food bank doing the best it could with the food it received from others, and believed that the food the food bank received was something that was beyond its control, and something that the service (like themselves) just had to accept, illustrated thus:

*You feel as if they are doing you a good favour, so you don't want to kick their ass, excuse my language. You can't turn round and tell them what to do because they are generous towards you. You feel as though if you try to say something to them that you would offend them.* P1

Yet when asked what they thought might improve their experience of the food bank service, almost all had various ideas about the types of food they felt should be given that would help them make better use of the food items received. Things that made it easier to cook food such as cooking oil were highlighted, as were items to make food taste better, or go further and last longer, such as salt and curry sauce. A few thought that getting more ‘eatable’ foods such as meat would also be a good idea. Having the ability to choose which items to put in their food bag was also regarded as something they would appreciate. In addition, there was some concern expressed that food banks were not meeting the needs of certain groups such as those with diabetes and families with children, and those who had difficulties transporting food home.

## 4 Discussion

This study has provided some insights into the lived experiences of those seeking emergency food aid in an affluent city in the north east of Scotland, and, the personal experiences and feelings of individuals who have had to seek help from others in a rich, consumer society like Scotland. It confirms some themes that have recently emerged about food aid use in the UK. Specifically, that disruptions to the incomes of those in receipt of social security benefits, are amongst the key drivers of emergency food aid seeking [6],[23].

But the findings also challenge some beliefs and emerging assumptions surrounding emergency food aid provision in general, and the behaviors and motivations of those seeking it. There is general tendency in countries like the UK and Scotland, to link individual's personal deficiencies and failures with ill-health, unemployment and homelessness, , including food poverty as opposed to the structural factors that underpin much of it [16]. From this perspective, benefit claimants are often framed as lacking general capabilities [24], including budgeting and cooking skills [8]. Yet, these findings suggest that considerable resources and skills are being employed by people in food crisis to mitigate their experience of food insecurity. This study also raises some questions regarding the health and wellbeing of those who are only currently seeking food aid in this country.

Analysis of the food banks database found that the primary users of the food bank were those in receipt of some form of government benefit. The qualitative interviews revealed that those individuals had only approached the food bank for help when they had experienced an unexpected and catastrophic drop in their normal weekly income. This sudden change in finances was commonly linked to some form of social security sanction or withdrawal. Those in this position had little or no savings to fall back on, and the food bank had represented a lifeline to their survival. This finding is consistent with recent English research that suggested that recent changes to the terms and conditions of social security entitlement is closely associated with increased numbers of people seeking help from organizations providing emergency food aid [25],[26]. In North America, changes to government social security policy over 20 years ago, has also been linked to the rise in emergency food aid use there [15],[27].

It was also apparent that chronic low income and unspecified degrees of indebtedness were prevalent features of participants' life circumstances. A recent UK study of indebtedness and food practices in low income households concluded that people rely on social security and/or chronically low incomes (described as ‘discipline of poverty’ by Dobson *et al.*, [28] had actually learned key life skills to make their limited resources go as far as possible, such as shopping more frequently with less money [29]. Managing to provide meals with very little has been reported elsewhere for low-income households in the UK [28]–[31]. It is conceivable therefore to imagine that a sudden and unexpected change in financial circumstances might overwhelm those individuals' capacity to pursue their normal, parsimonious food practices.

Our analysis also revealed that it is not just those from apparently chronically poor circumstances (i.e. who had been reliant of benefits long term) who had sought help. Participants who claimed to have been previously well off reported having to seek help from the food bank because of redundancy and ill health. The audit data indicated food bank clients had come from a variety of different locations in the city including from wealthy postcode areas, suggesting that people from a wide range of backgrounds had made use of this facility—not just those living on low incomes or social security. In this case, single men were the heaviest users of this particular food bank. This finding, of men being over represented in food bank use statistics, has also been found in a recent study that was conducted in the largest city in Scotland [32]. Yet, local anecdotal evidence also suggests that families with young children are more obviously using local food banks in some areas of the city, with elderly people using the local food bank more often in other areas. Moreover, the national picture of food bank use and it demographic characteristics is not clear [5].

Our participants' descriptions of food management, culinary skills and insights suggest they possessed considerable resilience and resourcefulness in managing the food items they received from the food bank; which is at odds with a more widely held view of people living in poverty and their behavioral deficits. This study found participants making significant efforts to manage their donated food. Stretching out donated food items to make them last as long as possible, i.e. over several days, was a key concern; as was using cooking techniques that required minimal amounts of fuel to prepare, and wasting as little food as possible. As Goode (2012) points out, these are precisely the behaviors that policy makers aspire we all pursue [29]. Furthermore, those living in temporary or hostel accommodation reported dealing with the additionally stressful challenges and uncertainties associated with having limited, secure food storage options, and access to suitable cooking facilities: which are amongst the most basic of prerequisites required to prepare and produce edible meals from food.

Tarasuk (2014 & 2002) and Dowler (2013) have both highlighted that policy responses associated with food poverty (found in the UK and in North America) are often grounded in misconceived beliefs about a widespread lack of food budgeting and cooking skills amongst the poor, which have very little empirical basis [8],[13],[33]. Our findings, i.e of highly food insecure people possessing food knowledge and skills, are also reflected in a study of very low-income mother's experiences of feeding the family [34], and in recent work that explored household budgetary practices of indebted families which found that considerable skill and multiple strategies had been used to manage the family income [29]. Indeed, Dowler and Lambie-Mumford (2015) have argued that food bank use is generally indicative of individuals having exhausted all other avenues, in the face of severe economic constraints, in their efforts to maintain their food security; such as cutting back, trading down on food quality, skipping meals and accepting gifts of food from friends and family [7]. Other researchers have similarly found that food insecure people often have sophisticated food budgeting, procurement and meal preparation knowledge; and devote considerable skill and effort to obtaining food for themselves and their families within the constraints of their household budgets [35]–[37].

It was striking (sadly so) that participants were not receiving food they normally ate or enjoyed from the food bank, but were taking what they were given. In addition, they all indicated general reluctance about asking staff for food items they liked. The fieldwork observations also confirmed this was the case beyond the interview data. Research into food bank operations in other jurisdictions has indicated that food aid recipients generally have little or no choice over what was given to them [27],[33]. Moreover, recent research in Canada and the UK has also established that food banks are constrained in what they can offer, by the very nature of individual public and corporate donations received [8].

The resigned lack of choice, coupled with effusive expressions of gratitude for the food and help they had received from the food bank staff, brought a profound sense of participants' disempowerment into sharp relief. In addition, their accounts of shame and desperation raises questions about food bank users' sense of well-being. For as Poppendieck (1999) has observed, relying on food charity and hand-outs undermines human well-being and basic dignity, and risks negation of a person's social and economic worth and value as consumers and family providers [27], and that, *“in our culture, with its stress on independence, .. is tantamount to an admission of failure”* (pg 240). Indeed, this “deficient” status may be acutely experienced in today's consumer society (38), and the experience of needing help with feeding as an adult, this most basic of human needs, may be particularly degrading (ibid). Van der Horst *et al.* (2014) recently explored the emotional experiences of food bank users in the Netherlands. They found that participants did not think their experiences of poverty were being taken seriously by the food bank volunteers, and felt that the “compulsory gratitude” they were expected to express in receipt of their food parcel, and, the experience of existing on food waste *“that would otherwise be given to pigs”*, were humiliating experiences [39]. Hamelin *et al.* (2009) found in their study of food aid assistance and poverty alleviation agencies, the perspectives of staff working for such agencies did not match the daily reality of the very low-income households they were helping. Researchers found a lack understanding of, and under-estimation about the impact of poverty on the lives of the people the study participants were purporting to help [35]. Beyond food bank research, it is also important to acknowledge existing and longstanding evidence that has found that the experience of lacking control over aspects of daily living, and powerlessness and recognition of one's low status in the prevailing social system, has a negative impact on individual health and well-being over the life course [40].

Furthermore, emergency food aid use is commonly viewed as a temporary/ transitory experience for most people. Yet, there is evidence that longer, sustained use is becoming the norm for an increasing number of people [8],[26],[39],[41]. This trend, coupled with these findings suggest there is an urgent need to conduct research into the effects and impacts of the experience of seeking and receiving food aid on mental and physical well-being in this country, as van Horst *et al.* (2014) in the Netherlands and Graithwaite *et al.* (2015) in the UK have also recently called for [39],[42].

These findings also raise questions about the emergent role of charitable organizations in the Scottish (and UK context) in addressing the growing problem of food poverty in this country [43]. For as George Kent has argued, *“dignity comes not from being fed but from being able to feed one's self”?*(Kent, 2005: pg 46). The need to have an informed debate about the preferred and accepted means of addressing this issue is particularly important at this critical juncture, given that it seems likely that further ‘austerity’ orientated public policy measures will be implemented by the newly elected UK government [44]; measures that are strongly implicated with food poverty [1],[23],[26]. At the same time, questions are currently being asked about the purpose of the state and of government in this [45]. Chilton and Rose (2009) argued that there is a prevalent belief in the US that food poverty and hunger should be solved by charitable, food-based solutions [46]. From a charitable perspective, responses to hunger and food poverty are grounded in notions of their being a ‘needs-based’ issue, and assumes that the needs of hungry people needs can be met by being fed by others. Programmers or policies that use needs-based approaches tend to do so without expectation of action by the recipient, but at the same time, offer no legal protections or obligations to those recipients. On the other hand, those advocating a ‘rights-based approach’ to food poverty provide a counter argument to this perspective. Rights-based approaches assert that access to safe, nutritious food is a basic human right, a requirement for human health, and the means by which it is possible to promote well-being and human dignity [15],[16],[43],[46],[47]. This perspective is underpinned by the assertion that governments (not charities) should provide the ‘enabling environments’ that support people to nourish themselves [16],[48].

These findings also point towards one further public health issue. One participant drew our attention to challenges he had personally experienced in meeting his nutritional needs from the food bank parcel he had received. In addition, it was clear most of those who took part in our study were suffering from some form of underlying health problem or problems; a finding that concurs with Parry *et al.*'s (2014) recent study of food bank users in England [26]. While, we did not establish whether those individuals believed their nutritional needs or dietary requirements were being met or not, some commented that the food bank offerings would be inadequate for people with specific conditions like diabetes, or for families with young children. While, it would be inappropriate to extrapolate these finding to all food banks, anecdotal evidence in Scotland suggests that some food bank operations are better able to respond to various nutritional and dietary requirements than others. However, relying on food aid as a regular means of sustenance, as has been documented elsewhere, represents a challenge for those affected by chronic illness. International evidence indicates that people affected by chronic health conditions and food insecurity find their ability to manage their condition effectively is further exacerbated by the variability of supply and often poor nutritional quality of the food distributed by the food bank ‘food system’ [15],[27],[49]–[51]. Furthermore, despite recent efforts in the US to make food bank offerings more healthful [51], international studies of food banks have indicated that the supply and quality of food available to them is insufficient to meet the nutritional needs of their clients, is unpredictable, and not within the control of individual food bank operations [8],[49],[52].

Finally, as highlighted above, in the absence of a direct measure of food poverty in the UK, emergency food aid use trends are increasingly regarded as an indication of an emerging public health crisis [3],[11],[12]. Yet evidence from other countries indicates that many food insecure individual or households do not, or are unable, to seek food aid. For example, in Canada, where household food insecurity data has been collected in routine health surveys since 2004, only 20–30% of food insecure households are actually using food banks [53],[54]. This raises similar questions here as to the possible extent to people who are food insecure in this country may or may not be using food banks, why they may not be doing so, and how such households not seeking food aid are managing. And, given the known limitations of, and challenges faced by food banks in addressing and responding to food insecurity [52], questions arise as to why they continue to persist and proliferate.

### Strengths and limitations of the study

There are some important limitations and practical issues to be aware of when interpreting the results of this work. Despite the fact that the second author had developed good relationships with many food bank users during four month participant observation period; in the end, only seven individuals agreed and felt comfortable enough to be formally interviewed, for the study. So those who agreed to participate were clearly self-selecting. From our point of view, this was not unexpected, (as we highlighted at the beginning of the article) but frustrating nevertheless, as a number of the themes formally presented here had also emerged during many daily informal conversations that took place over four months. However, this finding served to strengthen our understanding that the experience of food aid seeking is a shameful and difficult experience for the majority of people who do so. It is important to note, that those who agreed to participate in the interview study were representative of the demographic profile of the users of this particular food bank. In addition, this is but one study, of one food bank in a large urban centre in Scotland and we cannot claim these findings are generalizsable to all others. Nor can it claim to be representative of all people who are currently using food banks, or, who maybe food insecure but have chosen not to or are unable to access a food bank.

However, as we have highlighted here, some of the themes we found resonated with research that has been carried out in Northern America where food banks have an established history. This relatively small study has also yielded rich insights and contributed to a better understanding of (as called for by Sosenko and colleagues [4]) the lived experiences of an increasing number of people in Scotland who are, or are becoming marginalised by virtue of their economic and social disadvantage, and turning to food banks for help. In addition, this study has contributed to an under-researched area in the food insecurity literature. Pine and de Souza (2013) have argued that the food security scholarship has largely focused on food insecure individuals as objects of research study. They argued that there was a pressing need for more community-based participatory research, where those communities' perspectives and experiences were placed central to the research process and in the generation of data and ideas likely to yield more effective policy responses to address food poverty [55]. This research also provides, for those intent on advancing the cause of those living in poverty in this country, some experiential data upon which to critically consider the possible unintended, long-term consequences of high profile ‘food bank drives’, like that recently described by Martin Caraher in the aftermath of the recent Scottish independence referendum [56].

### Areas for future research

This work has highlighted a number of questions that require further investigation. These include the need to gain a better understanding of the range of people using Scotland's food banks at the current time. In the face of contemporary trends in food bank use, predicted rises commodity and utility prices due to climate change [57], and the proposed continuation of current government social and economic policy, a more detailed and comprehensive understanding of the range of nutritional and health needs of food insecure individuals and subgroups is required. This research should establish more clearly who is or might be at risk from food insecurity (including those who may not be turning to food banks for help), and consider monitoring for adverse health outcomes associated with food insecurity that have been established on other jurisdictions [58],[59]. Our study suggests that there are an unknown number of people with chronic health conditions seeking help from food banks, and raises questions about the impact repeated food bank use might have on the health and well-being of those affected. There is also an urgent need to conduct research into the impacts of the general experience of seeking and receiving food aid on mental and physical well-being in this country, given the rising numbers to people seeking help with feeding from charitable food sources. Moreover, given the apparent magnitude of the problem, and the dearth of research of the lived experiences of food insecurity in this country, there is also a need for research that more directly represents the lives of those affected by it in order to understand how those communities might mobilise themselves for food security and empowerment [55].

## 5 Conclusion

This study indicates that people who use food banks only do so after experiencing a severe financial shock, and are likely to be experiencing great shame and potentially health damaging emotional challenge in the process. Food aid clients in this study also reported considerable resourcefulness in managing their donated food items carefully, and with a view to doing so as efficiently as possible. This included adopting a range of strategies to minimize food wastage, despite the fact that the donated food they received did not always match their particular preferences or dietary requirements.

It is important to exercise care in the formulation of research and policy responses to ensure that they take account of and direct attention towards the structural as well as individual level determinants of food poverty [14]. Policy interventions that are designed on the basis of erroneous assumptions about the lived experiences, values and existing capabilities of the intended policy beneficiaries, may default to the ‘individual deficit’ conception of the problem, and underplay the extent to which structural level factors play a role in propagating and sustaining the policy problem (ibid). Insights gained from this study suggest that recent policy proposals to address food poverty in the UK, such as measures to increase the supply of commercial food waste to food banks, and increasing the numbers of food bank users participating in food-orientated education programmes, may be ineffective in addressing household food insecurity in the medium to longer term, without concomitant effort to implement public policy that can address material disadvantage.
